# Hypertrophic Cardiomyopathy Mutations of Troponin Reveal Details of Striated Muscle Regulation

**DOI:** 10.3389/fphys.2022.902079

**Published:** 2022-05-26

**Authors:** J. M. Chalovich, L. Zhu, D. Johnson

**Affiliations:** Department of Biochemistry and Molecular Biology, Brody School of Medicine, East Carolina University, Greenville, NC, United States

**Keywords:** cardiomyopathy, troponin, striated muscle, states of actin, troponin T, troponin I, acrylodan tropomyosin, muscle contraction regulation

## Abstract

Striated muscle contraction is inhibited by the actin associated proteins tropomyosin, troponin T, troponin I and troponin C. Binding of Ca^2+^ to troponin C relieves this inhibition by changing contacts among the regulatory components and ultimately repositioning tropomyosin on the actin filament creating a state that is permissive for contraction. Several lines of evidence suggest that there are three possible positions of tropomyosin on actin commonly called Blocked, Closed/Calcium and Open or Myosin states. These states are thought to correlate with different functional states of the contractile system: inactive-Ca^2+^-free, inactive-Ca^2+^-bound and active. The inactive-Ca^2+^-free state is highly occupied at low free Ca^2+^ levels. However, saturating Ca^2+^ produces a mixture of inactive and active states making study of the individual states difficult. Disease causing mutations of troponin, as well as phosphomimetic mutations change the stabilities of the states of the regulatory complex thus providing tools for studying individual states. Mutants of troponin are available to stabilize each of three structural states. Particular attention is given to the hypertrophic cardiomyopathy causing mutation, Δ14 of TnT, that is missing the last 14 C-terminal residues of cardiac troponin T. Removal of the basic residues in this region eliminates the inactive-Ca^2+^-free state. The major state occupied with Δ14 TnT at inactivating Ca^2+^ levels resembles the inactive-Ca^2+^-bound state in function and in displacement of TnI from actin-tropomyosin. Addition of Ca^2+^, with Δ14TnT, shifts the equilibrium between the inactive-Ca^2+^-bound and the active state to favor that latter state. These mutants suggest a unique role for the C-terminal region of Troponin T as a brake to limit Ca^2+^ activation.

## The Scope of Troponin Mutants

Naturally occurring mutants of troponin have been identified that stabilize each of the states of regulated actin. Those states of actin have different abilities to stimulate myosin ATPase activity. In addition to giving insight into muscle disorders, these mutants have provided tools to study normal muscle function and regulation. The use of troponin mutants as tools will be apparent in the first part of this document as we review concepts of regulation, the states of regulated actin and quantitation of those states. These concepts are essential as the mutants of troponin that we studied function by changing the distribution of those actin states.

## Regulation of Contraction

Muscle movement occurs as two filamentous protein complexes (myosin and actin) slide past each other in a process that is driven by the concentration gradient of ATP and the free energy of ATP hydrolysis. Actin binding to myosin greatly increases the rate of ATP hydrolysis by myosin; the conformational changes that occur during the cycle of ATP hydrolysis are coupled to force production. Cardiac and skeletal muscles are regulated primarily through modulation of the cofactor activity of actin through tropomyosin and troponin. This modulation may be fine-tuned by post translational modification such as phosphorylation of sites in TnI and TnT (Noland et al., 1989; Kobayashi and Solaro, 2005) and tropomyosin (Rao et al., 2009). Additional regulation may occur *via* C protein (Harris et al., 2004) or nebulin (Root and Wang, 2001). Phosphorylation of myosin light chains plays a modulatory role even in striated muscle (Sweeney and Stull, 1990). Here we limit our comments to regulation that occurs through the actin binding proteins tropomyosin and troponin.

### Actin Based Regulation

**FIGURE 1:**
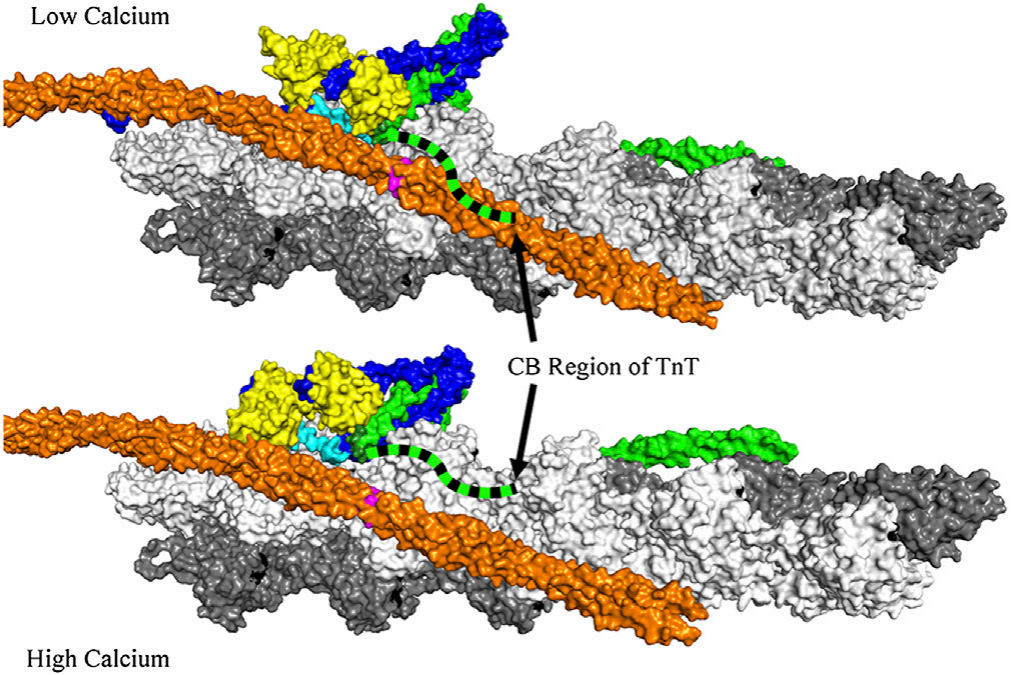
View of actin (grey and white), tropomyosin (orange), TnT (green), TnC (yellow) and TnI (blue) with the inhibitory region of TnI (light blue). Also shown are Cys 190 of tropomyosin (pink) and Cys 374 of actin (black). The coordinates for this figure were taken from PDB deposition 6kn7 (low calcium) and 6kn8 (high calcium) (Yamada et al., 2020). Superimposed on this figure is a possible location of the C-terminal basic region of TnT (green dashed ribbon). This ribbon extends from the end of the IT helix. Both the inhibitory region of TnI and the C-terminal basic region of TnT move away from actin-tropomyosin in the Ca^2+^-bound state. Actin filaments containing HAHA TnT at low free Ca^2+^ would resemble the wild type at high Ca^2+^ in some respects (see text). Note that the tropomyosin and troponin on the opposite face of the actin filament are not shown for clarity.

Actin filaments from striated mammalian muscle have tropomyosin bound along the length of both sides of the double actin helix (Figure 1). Each tropomyosin molecule covers seven actin protomers. A troponin complex is bound to each tropomyosin. The troponin complex contains 3 subunits: TnT (tropomyosin binding), TnI (inhibitory) and TnC (calcium binding). The inhibitory region of TnI and the C-terminal region of TnI remain bound to actin, in the relaxed state, resulting in positioning tropomyosin on the outer edge of the actin helical groove (intersection of the white and grey actin strands). Ca^2+^ binding to TnC opens a hydrophobic patch to which the switch region of TnI can bind (Herzberg and James, 1985) thus moving TnI away from actin (see the light blue region in Figure 1) and permitting tropomyosin to exist in an equilibrium between an inactive state and an active state that can support high ATPase activity and movement.

### The Role of Calcium and Force Producing Crossbridge Attachment

Calcium alone increases the k_cat_ for ATPase activity by about 18-fold and decreases the K_M_ for actin by about 2-fold, giving about a 36-fold activation at low actin concentrations (Chalovich and Eisenberg, 1982; El-Saleh and Potter, 1985). Full activation occurs when force producing myosin crossbridges accumulate and bind to actin and fix tropomyosin in the active state. The ATPase rate can be increased a further 4-fold or more by allowing binding of “activating” or force producing-like myosin subfragments to actin such as occurs when myosin subfragment-1 has bound ADP. We will describe this in more detail in the following section.

Initial observations of the activating effects of force producing crossbridges were at low ATP levels where myosin-ADP levels could rise and stabilize the active state of regulated actin filaments. Limiting experimentation to very low ATP concentrations precluded some questions of physiological significance as physiological levels of ATP are high relative to ADP. For example, determining the effects of the regulatory proteins on the binding of myosin to actin or on the kinetic constants that define actin activated ATP hydrolysis during contraction must be measured at saturating concentrations of ATP.

### Activation With N-Ethylmaleimide Labeled S1 (NEM-S1)

One way to study the role of both Ca^2+^ and “force producing” crossbridges, under saturating ATP conditions, is to dope the system with myosin or myosin subfragments that were treated with the sulfhydryl alkylating reagent N-ethylmaleimide (NEM). S1 that is extensively modified with NEM binds with high affinity to regulated actin even at saturating ATP levels and is able to activate regulated actin under those conditions (Pemrick and Weber, 1976; Meeusen and Cande, 1979; Heeley et al., 2006).

The activating effect of NEM-S1 does not stem directly from its high affinity but results from a change in the way that some chemical forms of S1, like NEM-S1, bind to actin as the free Ca^2+^ level increases. NEM-S1 binds more tightly to regulated actin at high free Ca^2+^ levels whereas S1-ATP binds with about the same affinity irrespective of the free Ca^2+^ level (Chalovich and Eisenberg, 1982; Kraft et al., 1995). Furthermore, increasing saturation of regulated actin with the stable S1-ATP analogue, pPDM-S1-ATP, at low ionic strength is not cooperative (Chalovich et al., 1983). However, S1-AMP-PNP binding to regulated actin is cooperative over the same level of saturation of actin sites even with the affinity of binding adjusted to the same level by using a higher salt concentration (Chalovich et al., 1983). Cooperativity occurs because of a transition from a low affinity state to a high affinity state of the regulated actin. S1-ATP and pPDM-S1-ATP bind with a similar affinity to both actin states so no stabilization of the high affinity state of actin occurs. Thus, we often distinguish chemical and nucleotide states of myosin as either being “activating” or “non-activating.” S1-AMP-PNP, S1-ADP, NEM-S1 and rigor S1 are activating while S1-ATP, S1-ADP-Pi and pPDM-S1-ATP are non-activating states. More will be said about this cooperative transition in the discussion of mutations that increase activation at all Ca^2+^ levels.

The additional activation that NEM-S1 provides over activation by Ca^2+^ occurs largely because of a decrease in the K_M_ for actin (Williams et al., 1988). Note that the K_M_ is the actin concentration required to reach ½ of the V_max_ and should not be confused with binding affinity. The affinity of unmodified S1-ATP for regulated actin was not appreciably increased by the presence of activating levels of NEM-S1.

It is interesting that the combination of two cardiomyopathy associated mutants, Δ14-TnT and A8V-TnC, together yield full activation by Ca^2+^ without the need of high affinity attachment of myosin to actin (Baxley et al., 2017). This is a clue that activation by force producing crossbridge binding and by mutations of troponin may share a common mechanism. The C-terminal region of TnT is essential for the ability of activating crossbridges to increase contractile activity.

## Disorders of Skeletal and Cardiac Muscle Regulatiory Proteins

Many naturally occurring mutants of tropomyosin and troponin produce defects in regulation that lead to contractile dysfunction. Some mutations affect protein folding or binding affinity leaving some actin units unregulated. Other mutations create regulatory complexes that have: 1) decreased activation at all Ca^2+^ levels 2) decreased activation at saturating Ca^2+^ but increased activation at low free Ca^2+^ or 3) increased activation at all Ca^2+^ levels. Several examples of these three classes have altered equilibrium constants among the inactive and active states of regulated actin (Chalovich, 2012; Lehrer and Geeves, 2014). Altering the distribution among the states of regulated actin is reasonable as it is troponin that stabilizes tropomyosin into the various positions on actin that define the states.

Our survey of mutations of TnT and TnI showed that five TnT mutants, associated with dilated cardiomyopathy, have decreased Ca^2+^ sensitivity. It is likely that these mutations stabilize the inactive states of regulated actin. Fifteen mutants of TnT and six mutants of TnI, associated with hypertrophic cardiomyopathy, have increased Ca^2+^ sensitivity; those mutants likely stabilize the active state. The association between the effect of mutations on Ca^2+^-sensitivity and the type of disorder has been previously noted (Robinson et al., 2002), (Robinson et al., 2007). Several mutations of TnI had a mixed effect on Ca^2+^ sensitivity that could be explained by stabilization of an inactive intermediate state. Before exploring these cases in detail, it is necessary to discuss the states of regulated actin and how they are measured.

### States of Regulated Actin

Many studies support the idea that troponin controls the ability of actin to accelerate myosin ATPase activity by changing the position of tropomyosin on actin (Huxley, 1972; Haselgrove, 1972); Parry and Squire, 1973; Wakabayashi et al., 1975; Pirani et al., 2005). Although the regulation of ATPase activity can be described by two states (inactive and active) (Hill et al., 1981), there appear to be three structurally distinct positions of tropomyosin on actin separated by relatively low energy barriers (Poole et al., 2006; Risi et al., 2017). Two of these three structural states are degenerate in terms of their inability to support rapid ATP hydrolysis by myosin (Johnson et al., 2016). The merits of two state (Heeley et al., 2019) vs. 3 state (Geeves et al., 2019) models have recently been discussed.

A 1981 model described two classes of states: 1 (inactive) and 2 (active). State 1 had two substates: one without Ca^2+^ and the other with bound Ca^2+^. A later model (McKillop and Geeves, 1993) described three states: blocked (B), closed or calcium (C) and either O (open) or M (myosin stabilized). Although the models differ in some fundamental respects, the states appear to be equivalent. Others make a further distinction between the active state stabilized by Ca^2+^ or by rigor myosin binding (Lehrer and Geeves, 2014). The ATPase activity of myosin is proportional to the fraction of actin in the active state. When myosin-linked regulation is present, the rate is also proportional to the fraction of myosin in the active state. We will not deal with myosin-linked regulation here.

### Determining the Fraction of Each Actin State

Quantitation of the active state is most confidently done by measuring a relative activity such as ATPase activity. The fraction of actin in the active state, F_A_, is then given by (v_obs_—v_min_)/(v_max_—v_min_) where v_obs_ is the measured ATPase rate, v_min_ is 0.67* the rate with zero calcium added and in the presence of EGTA, v_max_ is the rate measured at saturating Ca^2+^ and NEM-S1 and at the same actin concentration used in the other measurements. The term 0.67 comes from the rate of ATPase activity with a phosphomimetic mutant of TnI having Glu replacing the 3 phosphorylatable residues (Mathur et al., 2008). This lower rate may better reflect the inactive state. The value of v_max_ can be estimated by stabilizing the active state with both Ca^2+^ and activating or force-producing type crossbridges. The use of NEM-S1 to stabilize the active state (mentioned earlier) may involve rather large corrections (Baxley et al., 2017). By using Δ14-TnT, F_A_ is increased from 0.25 to 0.7 with Ca^2+^ alone. Thus, small amounts of NEM-S1 are sufficient to reach full activation and the corrections are minimized. The combination of Δ14-TnT and A8V-TnC gives F_A_ = 1 without the need of NEM-S1 at all (Baxley et al., 2017).

Actin filaments in the two inactive states can be distinguished based on differences in the rate of non-ATP containing myosin S1 binding to regulated actin in the presence and absence of Ca^2+^ (Trybus and Taylor, 1980; McKillop and Geeves, 1993), by the use of environmentally sensitive probes on tropomyosin (Ishii and Lehrer, 1993; Gafurov and Chalovich, 2007; Borrego-Diaz and Chalovich, 2010) and by FRET between different proteins of the thin filament (Shitaka et al., 2004; Shitaka et al., 2005; Johnson et al., 2019). However, there is uncertainty in quantitating the relative populations of the two inactive states because we do not know the values of these parameters when actin is 100% in each of the two inactive states. That is, at low Ca^2+^ there is a mixture of the two inactive states while at saturating Ca^2+^ there is a mixture of the two Ca^2+^-bound states, one inactive and one active.

One approach to determining the fraction of each of the inactive states is to use a model of regulation to calculate the values based on a series of assumptions. This approach was taken for estimating the state distribution based on the kinetics of ATP-free S1 binding to regulated actin (McKillop and Geeves, 1993). In that study, the values of the parameters K_b_, K_2_ and n were fixed at what were seen to be reasonable values, the rate of binding to the inactive-Ca^2+^-free state (B state) was set to zero and the rate of binding to actin in the presence of saturating Ca^2+^ (a mixture of the C and O states) was assumed to be at its maximum value. Although useful, such estimates are model dependent. Relaxing the restrictions led to different estimates of the actin distributions (Gafurov et al., 2004a). Furthermore, the assumption that Ca^2+^ is sufficient to produce the maximum rate of binding does not appear to be true with cardiac regulatory proteins (Gafurov et al., 2004a).

While current measurements of actin state distributions are imperfect, these methods accurately report changes in the distribution of states. These methods are also invaluable for measuring the kinetics of state transitions (Shitaka et al., 2005; McKillop and Geeves, 1993; Borrego-Diaz and Chalovich, 2010; Johnson et al., 2019).

## Troponin Mutants Serve as Benchmarks of Actin States and Demonstrate Additional Complexities of Regulation

Because some troponin mutants stabilize a particular state of actin, they can be used to calibrate measurements of actin state distributions. The S45E mutation of TnI gives a better estimate of the inactive amplitude as it gives a signal for acrylodan tropomyosin fluorescence 1.3x the wild type amplitude (Franklin et al., 2012). It is yet unclear if this represents 100% occupancy of the inactive-Ca^2+^-free state. Stabilization of a state that is functionally similar to the inactive-Ca^2+^-bound state can be achieved using Δ14-TnT mutant at low Ca^2+^ levels. The fully active state can be stabilized with the combination of A8V TnC and Δ14 TnT. A description of these and other mutants is given below.

### Mutations That Decrease Activation at all Ca^2+^ Levels

The example that we give here is not that of a disease-causing mutation but of the simulation of a normal regulatory event. PKC pseudo-phosphorylation of TnI at Ser-43, Ser-45 and Thr-144 (mouse sequence) reduces Ca^2+^ sensitivity and ATPase activity (Burkart et al., 2003). Actin filaments containing TnI with Glu inserted at positions 45, 43 and 45 or 43, 45 and 144 all had reduced ATPase activities which were especially pronounced at saturating Ca^2+^ (Mathur et al., 2008). The rates at saturating Ca^2+^ approached the same maximum limit as wild type upon activation with NEM-S1. Thus, the decreased rate was likely due to stabilization of the inactive state of regulated actin. The S45E mutant had a rate 0.89 times wild type while the 43/45/144 mutant was even lower at 0.67 times the wild type rate. Our working assumption is that the minimum value of ATPase activity, at low free Ca^2+^ is 0.67x the wild type rate. As already stated, the amplitude of acrylodan-tropomyosin fluorescence of S45E-TnI was 1.3x that of wild type actin filaments. Together with the lower ATPase activity observed with S45E-TnI, it is clear that wild type troponin does not fully stabilize the inactive-Ca^2+^-free state.

The idea that these phosphomimetic mutants stabilize the inactive-Ca^2+^-free state was reinforced by comparing the equilibrium binding of S1-ADP to actin filaments containing wild type and mutant TnI. Binding to actin filaments containing mutant TnI had a more sigmoidal cooperative binding curve than did wild type actin filaments. Increasing the fraction of actin filaments in the low affinity state has the effect of exaggerating the cooperative transition. The affinity of regulated actin containing the 45 and 43/45/144 mutants for Ca^2+^ was also decreased as expected for stabilization of the inactive state. Thus, it appeared that these phosphomimetic mutants of TnI shifted the equilibrium constants among the states to favor the inactive state.

### Mutations That Decrease Activation at Saturating Ca^2+^ but Increase Activation at Low Free Ca^2+^: Stabilization of an Inactive Intermediate State

Several missense mutations of TnI are associated with hypertrophic cardiomyopathy (Kimura et al., 1997). The R146G mutations were shown to reduce regulation with an elevated fiber force at low free Ca^2+^ levels and a depressed force at saturating Ca^2+^ (Lang et al., 2002). These TnI mutants increased Ca^2+^ sensitivity. The D191H, R146G and R146W mutations of mouse TnI stabilized the active state at low free Ca^2+^ and the inactive state at saturating Ca^2+^ (Mathur et al., 2009). The effects of R146G-TnI could not be attributed to a change in affinity for actin-tropomyosin. We proposed that these mutations of TnI stabilized a form of regulated actin that was between the inactive and active states (Mathur et al., 2009).

The intermediate state of regulated actin that was stabilized by R146G-TnI was inactive toward stimulating myosin catalyzed ATPase activity (Mathur et al., 2009). This state had been assumed to be inactive in models of regulation of ATPase activity (Hill et al., 1981) and of myosin S1 binding to regulated actin (McKillop and Geeves, 1993).

R146G and R146W occur within the inhibitory region of TnI that is essential for forming the inactive-Ca^2+^-free state. The inhibitory region (Tao et al., 1990) and the COOH terminal mobile region of TnI (Tripet et al., 1997) are bound to actin in the inactive-Ca^2+^-free state and are released at saturating Ca^2+^ levels. Other residues within the inhibitory region of TnI are likely to have an equal or greater effect on activity than the R146 mutations (Van Eyk and Hodges, 1988). Elimination of the inhibitory region of TnI is sufficient to eliminate the inactive-Ca^2+^-free state. However, the inhibitory and COOH regions of TnI are insufficient to form the inactive- Ca^2+^-free state of regulated actin. Rather, the C-terminal basic region of TnT is also required as shown later in this document.

### Mutations That Increase Activation at all Ca^2+^ Levels

Hypertrophic cardiomyopathy is most often associated with those mutations of troponin that cause an increase in Ca^2+^ activation (Robinson et al., 2007). These mutants are characterized by increased sensitivity to Ca^2+^ in muscle fiber preparations and an increase in actin-activated ATPase activity in solution studies. We have focused our attention on one TnT mutation that gives the largest increase in activity that we are aware of, that is Δ14-TnT.

Two splice variants of TnT were identified in a family that resulted in a deletion of the last 14 amino acids of TnT (Δ14-TnT) in one case and that replaced the last 28 amino acids with a novel stretch of 7 amino acids in the other case (Thierfelder et al., 1994). Muscle fibers having Δ14-TnT had an increased activity (Nakaura et al., 1999) and had an increased response to both Ca^2+^ and to high affinity, ATP-free myosin binding (Stelzer et al., 2004). The actin activated ATPase activity produced with actin filaments containing Δ14-TnT was higher than that with wild type filaments at both low and high Ca^2+^ concentrations (Gafurov et al., 2004b; Baxley et al., 2017) provided that the actin concentration was sub-saturating. The rates of wild type filaments and those containing Δ14-TnT were also the same when maximum activation was achieved by the addition of NEM-S1 (Gafurov et al., 2004b; Baxley et al., 2017).

Some hypertrophic cardiomyopathy mutations of TnC also moderately enhance Ca^2+^ activation of ATPase activity and sensitize fibers to Ca^2+^ (Landstrom et al., 2008). Whereas Δ14-TnT increased the actin activated ATPase activity by 2.2-fold, A8V-TnC increased the rate by 1.8-fold (Baxley et al., 2017). The combined effects of A8V-TnC and Δ14-TnT increased the ATPase activity 3.2-fold giving a rate identical to that of wild type filaments that were fully activated with Ca^2+^ and NEM-S1 (Baxley et al., 2017). These results suggest that troponin has the inherent ability to stabilize the active state of actin (Baxley et al., 2017) as well as the more well-known ability to stabilize the inactive state at low Ca^2+^ levels. The basic C-terminal region of TnT attenuates the stabilization of the active state that would otherwise occur at saturating Ca^2+^. Higher animals having the CB region have gained a dual activation system requiring Ca^2+^ and force producing crossbridges (Johnson et al., 2020).

The ATPase rates used to estimate the activation by Ca^2+^ were measured at low actin concentrations so that the values obtained were proportional to k_cat_/K_M_. When ATPase rates were measured with increasing concentrations of regulated actin, the values with Δ14-TnT-A8V-TnC and with actin in the absence of tropomyosin or troponin approached the same maximum rate. Troponin-tropomyosin increases the effectiveness of actin as a cofactor in accelerating ATPase activity by decreasing the K_M_, that is the concentration of regulated actin required to reach half of the maximum rate. This agrees with an earlier study that used NEM-S1 to fully activate the system (Williams et al., 1988). That study showed that the decrease in K_M_ occurred without a change in the affinity of S1-ATP for regulated actin.

This brings up a point that is incompletely understood. Ca^2+^ increases the ATPase activity of regulated actin primarily by increasing the k_cat_, with little change in K_M_ or in affinity of S1-ATP for actin (Chalovich and Eisenberg, 1982). Reaching full activation by using NEM-S1 or Δ14-TnT and A8V-TnC increases the rate another 3-4-fold and that increase occurs primarily with a decrease in K_M_ with a smaller change in k_cat_ and no appreciable change in S1-ATP affinity for actin (Williams et al., 1988). If these increases in rate are due only to the increase in the population of actin in the active state, then one would have expected full activation to also be driven primarily by an increase in k_cat_.

Further evidence that the Δ14 mutant of TnT stabilizes the active state of regulated actin came from studies of equilibrium binding of myosin S1 to regulated actin. The affinity and shape of the binding curves are dependent on the free Ca^2+^ level, ionic strength and nucleotide bound to S1 (Greene and Eisenberg, 1980), (Chalovich et al., 1983). At low free Ca^2+^ levels, binding of species such as S1-AMPPNP, S1-ADP and rigor S1 increases in a sigmoidal fashion as the free concentration of S1 increases. This occurs, as was stated earlier, because the regulated actin exists in an equilibrium between a lower affinity form and a higher affinity form for binding of non-ATP containing species of S1 with the lower affinity form being favored at low free Ca^2+^ (Hill et al., 1980). The binding of activating species of S1 stabilizes the higher affinity form of regulated actin in a cooperative manner. Binding is characterized by the equilibrium constant between the two binding configurations of regulated actin, the cooperativity of the transition and the affinities of the low and high affinity states for actin (Hill et al., 1980).

Replacing Δ14-TnT for wild type TnT made the binding in the virtual absence of Ca^2+^ become more similar to that observed at saturating Ca^2+^ (Gafurov et al., 2004b). Removing the C-terminal 14 amino acid residues from human cardiac TnT stabilized the high affinity configuration of actin over a range of ionic strengths (L’ increased in the Hill model). That is, the C-terminal region of TnT stabilized the low affinity binding state of regulated actin.

### HAHA-TnT and Other Constructs of Troponin T for the Study of Regulation

Just as NEM-S1 was created as a tool for studying activation by “activating” myosin species, HAHA-TnT was designed to permit study of the involvement of TnT in that activation. HAHA stands for High Ala, High Activity. HAHA-TnT was created by replacing the Lys and Arg residues in the CB region of cardiac or skeletal TnT (in bold in Table 1) with Ala. This produces a TnT that has effects on regulation much like those of Δ14-TnT without eliminating the entire C-terminal region (Johnson et al., 2019). We also produced 289C-HAHA-TnT that has an added C-terminal Cys residue that can incorporate reporter groups. The wild type control for the 289C-HAHA-TnT is 289C-TnT. Reporter groups have also been placed on 275C of both HAHA and wild type TnT.

**TABLE 1:** Mammalian C-terminal troponin T sequences compared with the C-terminal TnI sequence.

Troponin type	C-terminal
Cardiac TnT	QKVSKTRGKAKVTGRWK
Fast Skeletal TnT	QKHSKKAGTPAKGKVGGRWK
Slow Skeletal TnT	QKFRKGAGKGRVGGRWK
Cardiac TnI	RGKFKRPTLRRVRISAD 152

### Actin Filaments Containing Δ14-TnT or HAHA-TnT do Not Occupy the Inactive-Ca^2+^-free State in the Virtual Absence of Ca^2+^

Wild type regulated actin filaments containing acrylodan-labeled tropomyosin show an increase in fluorescence when they progress to the inactive-Ca^2+^-free state. There was no such fluorescence change with regulated actin containing either Δ14-TnT (Franklin et al., 2012), or 289C-HAHA-TnT (Johnson et al., 2019) indicating that the inactive-Ca^2+^-free state (or B state) was not formed.

The S45E mutant of TnI that stabilizes the inactive-Ca^2+^-free state and gives a 30% enhancement of the acrylodan-tropomyosin signal did not rescue the total absence of signal with Δ14-TnT (Franklin et al., 2012). The lack of additivity suggests that the C-terminal region of TnT is essential for forming the inactive-Ca^2+^-free state. We predict from these observations that the CB region of TnT stabilizes the binding of the switch and C-terminal regions of TnI to the actin filament. That hypothesis is supported by FRET measurements between these regions of TnI and both actin-374 and tropomyosin-190 (Zhu et al., 2022).

## Determination of Functional Regions Within the C-Terminus of TnT

Both the N- and C-terminal regions of TnT have regulatory roles. The N-terminal region of TnT is isoform specific and alters the properties, such as Ca^2+^ sensitivity, to specific muscle types (Jin, 2016). The constancy of the C-terminal region suggests that it functions on a more basic level to maintain normal regulation. The CB region of TnT does vary among animal species and that may give clues to its function.

The C-terminal region of TnT from mammals shows a preponderance of basic residues and a terminal sequence of GRWK (Table 1). The inhibitory region of TnI is also highly basic and there is a suggestion of an evolutionary link between these regions (Brunet et al., 2014). Such patches of basic residues may indicate that electrostatic interactions involving the C-terminal region of TnT may be important in its observed functions.

**FIGURE 2:**
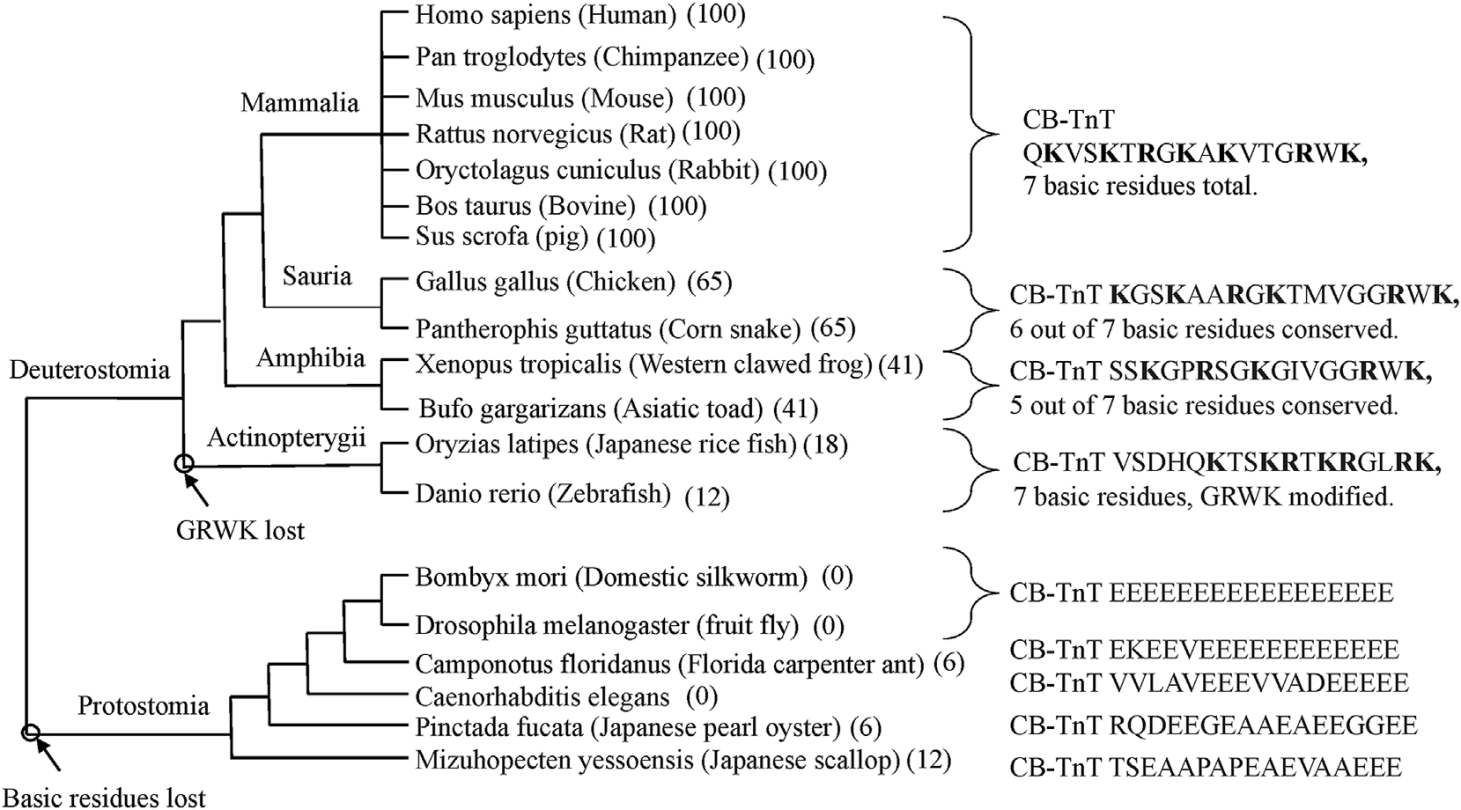
Cladogram of the last 17 residues of cardiac TnT. The mammalian sequences are all identical. They contain seven basic residues within this 17 residue stretch and have a terminal GRWK. Sauria have 65% sequence conservation with 6 basic residues and a terminal GRWK. Amphibia are 41% conserved with five basic residues and a terminal GRWK. Actinopterygii have 18% sequence conservation but have seven basic residues with the terminal sequence GLRK. Protostomia lack the C-terminal basic region altogether. Note that the Protostomia have the same type of TnT in all striated muscles.

Figure 2 shows that the CB region of TnT is highly conserved in cardiac muscle of mammals. Reptiles, birds, amphibians, and fish have different C-terminal sequences but maintain a large number of basic residues. Based on the results of stepwise truncation of residues from the C-terminal of cardiac TnT, the limitation of Ca^2+^ activation is proportional to the fraction of basic residues conserved (Johnson et al., 2018). Some fish TnT have a C-terminus that is highly basic while lacking the terminal GRWK.

The basic C-terminal region is absent in Protostomia. Some members of Protostomia have a long stretch of Glu residues at their C-terminal. There is evidence that this acidic region is a Ca^2+^ buffer that assists the high frequency oscillations of insect flight muscle (Cao et al., 2020). It is interesting, in this regard, that the flight muscle of some birds also has a long stretch of Glu residues but at the N-terminal region (Jin et al., 2008). It will be interesting to see how the regulatory properties of avian and mammalian TnT compare.

### Mutants of TnT Demonstrate the Importance of Overall Positive Charge of the CB Region

**FIGURE 3:**
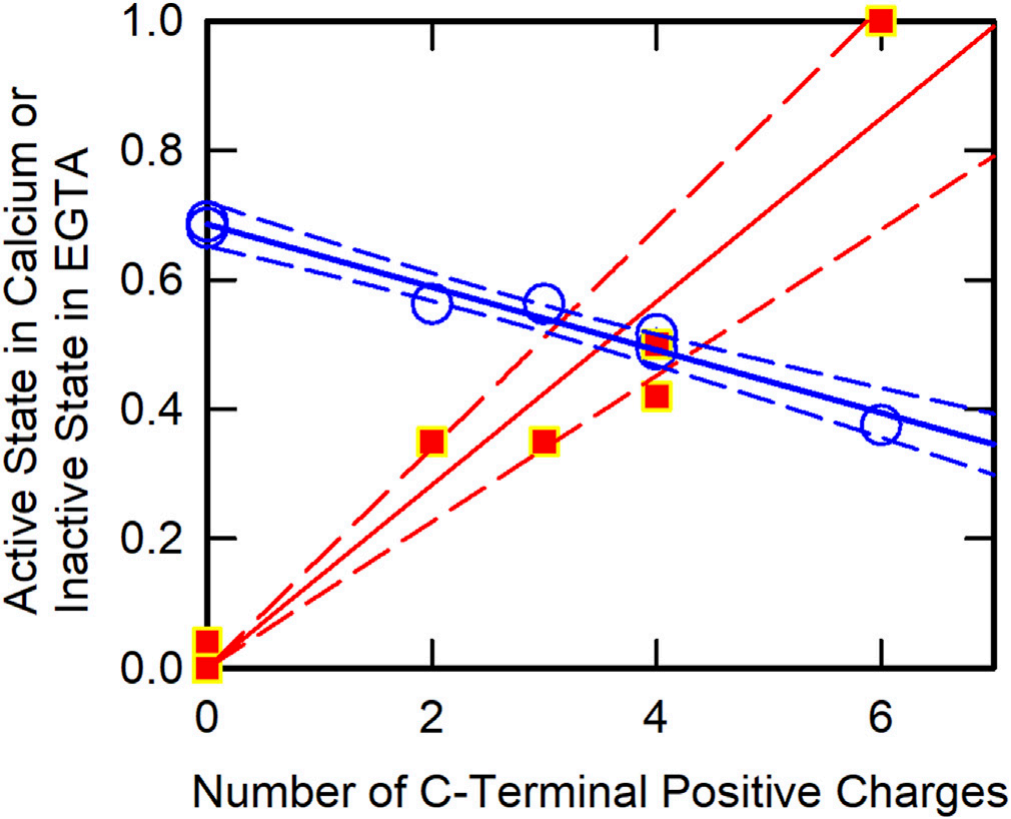
C-Terminal TnT Charge Effects on Actin Filament States. The fraction of actin in the active state at saturating Ca^2+^ was determined by ATPase assays (green circles). The fraction of actin in the inactive state in the virtual absence of Ca^2+^ was determined by acrylodan tropomyosin fluorescence (red squares). Data were obtained from earlier publications (Johnson et al., 2018), (Johnson et al., 2019) with linear least square fits (solid lines) and 95% confidence limits (broken lines).

To identify key residues within the CB region of TnT, we made a series of C-terminal truncation mutants. Figure 3 shows the approximate fraction of regulated actin in the active state and in the inactive-Ca^2+^-free state as a function of the number of deleted positive charges. At saturating Ca^2+^, the fraction of regulated actin in the active state increased in roughly a linear fashion as the CB region was shortened. Likewise, the fraction of regulated actin in the inactive-Ca^2+^-free state decreased in a linear manner as the number of positive charges was reduced. These data show that the basic residues are responsible for stabilizing the inactive state at low Ca^2+^ and for destabilizing the active state at saturating Ca^2+^. That the loss of positive charges from the C-terminal region of TnT can produce an activating effect has also been shown with the R278C mutation (Morimoto et al., 1999), (Szczesna et al., 2000), the K273E mutation (Venkatraman et al., 2003), (Messer et al., 2016) and the K280N mutation (Messer et al., 2016).

The rate of rigor S1 binding to regulated actin containing wild type cardiac troponin was about 4-fold faster at high Ca^2+^ than at low free Ca^2+^ indicating that the C and M states (inactive-Ca^2+^-bound and active states) permit faster binding (Trybus and Taylor, 1980), (McKillop and Geeves, 1993). The rate of binding of rigor S1 to actin filaments containing 289C-HAHA at low Ca^2+^ was similar to that observed with wild type regulated actin at saturating Ca^2+^. That is, removal of the C-terminal basic residues of TnT increased the occupancy of these faster binding states. It was also interesting that the rate of binding of rigor S1 to 289C-HAHA containing actin filaments at saturating Ca^2+^ was 1.8x that of wild type at saturating Ca^2+^. That is, with cardiac troponin, the rate of binding to actin in the active state (M state) is faster than to actin in the inactive-Ca^2+^-bound state (C state) (Johnson et al., 2019). That difference was not observed in the case of skeletal troponin (Lopez Davila et al., 2020).

The HAHA mutant of TnT had other similarities to Δ14 TnT. For example, the acrylodan-tropomyosin fluorescence assay failed to detect evidence of the B state in the virtual absence of Ca^2+^ (Johnson et al., 2019). However, the ATPase activity remained very low indicating that the active state was not populated. We concluded that actin filaments containing HAHA TnT exist in a state that is functionally like the C (inactive-Ca^2+^-bound) state even at low free Ca^2+^.

HAHA TnT increased the ATPase activity at saturating Ca^2+^ over values obtained with wild type actin filaments. Placing 289C-HAHA TnT into a cardiac fiber preparation increased the Ca^2+^ sensitivity by 0.24 pCa units. Overall, HAHA TnT behaved similarly to Δ14 TnT.

The Cys residues engineered into the CB region of TnT made it possible to follow changes in this region. The transition from the inactive-Ca^2+^-bound state of wild type actin filaments to the inactive-Ca^2+^-free state was accompanied by movement of TnT-289 toward tropomyosin-190 (Johnson et al., 2019). Furthermore, the apparent rate constant of this transition was similar to the rate constant for acrylodan tropomyosin fluorescence over a range of temperatures. When the same study was done with 289C-HAHA-TnT there was no change in FRET as removal of the basic residues made it virtually impossible to occupy the inactive-Ca^2+^-free state.

### Muscle Fiber Studies Confirm the Role of Positively Charged Residues Within the CB Region of TnT

The previous solution studies raise an interesting question of how muscle contraction would change if there were no B state and if Ca^2+^ gave ≥ 3-fold greater activation of ATPase activity than normally observed. This question was examined with the *in vitro* motility assay and with a cardiac fiber preparation (Johnson et al., 2020).

Actin filaments containing Δ14-TnT had an enhanced rate of movement at both low and high Ca^2+^ levels (Johnson et al., 2020). The maximum speed of movement was about 1.8x faster than wild type at saturating Ca^2+^. There was no movement of wild type filaments at low Ca^2+^ but Δ14-TnT-containing filaments moved at 13% of the maximum observed rate. No change in Ca^2+^ sensitivity was observed.

Cardiac muscle fiber preparations containing Δ14-TnT produced the same maximum force as fibers with wild type troponin and had an increased Ca^2+^ sensitivity by 0.2 pCa units (Gafurov et al., 2004b). Fibers containing both Δ14-TnT and A8V-TnC produced about 18% more force than fibers with wild type troponin (Johnson et al., 2020). Both Δ14-TnT and the combination of Δ14-TnT & A8V-TnC increased the basal activity by about 3-fold and increased the Ca^2+^ sensitivity by 0.3–0.34 pCa units.

Taken together, these results show that eliminating the inactive-Ca^2+^-free state results in increased basal activity. The force produced by fiber preparations increased at all Ca^2+^ levels. The results of the solution assays and the *in vitro* motility assay were very similar; in both cases the loads were small as were the number of attached myosin molecules. Muscle fiber preparations had greater activation at low Ca^2+^ levels but did not have large increases, over wild type, in maximally activated force.

Many of these results were recapitulated in skeletal troponin-tropomyosin using a skeletal version 251C-HAHA-TnT or Δ16 skeletal TnT (Lopez Davila et al., 2020). Effects of the loss of charges at the C-terminal region of skeletal TnT included increased ATPase activity at low and high Ca^2+^, loss of the acrylodan tropomyosin fluorescence increase in transitioning to the B state, and an increase in Ca^2+^ sensitivity by 0.8–1.2 pCa units. Skeletal fibers containing the mutant TnT varieties produced force even at pCa 7.5 where fibers are normally relaxed. The C-terminal basic region of skeletal TnT, like that of cardiac TnT, is required for forming the inactive-Ca^2+^-free state and for limiting the activation by Ca^2+^.

## Troponin Structure Alteration by Mutations

Regulation of mammalian striated muscle contraction involves alterations in protein-protein contacts among TnC, TnI, TnT, tropomyosin and actin. Because the CB region of TnT causes large changes in the response to Ca^2+^, it seems likely that substantial changes in these protein-protein associations also occur. We investigated Ca^2+^-dependent changes in the CB region of TnT and the effect that the CB region has on the well documented changes in TnI contacts that occur during activation. The CB region is natively unstructured and is invisible in high resolution structures of troponin. We utilized Förster Resonance Energy Transfer (FRET) to monitor changes in the CB region of TnT and to map the changes that the CB region of TnT produces in other key regions of troponin.

Readers may wish to consult more detailed descriptions of troponin structure than are present in this manuscript. A detailed description of the structure of a large part of the troponin complex is available (Takeda et al., 2003). Most recently, the structure of much of regulated actin complex has been published (Yamada et al., 2020) along with a helpful commentary on that structure (Tobacman, 2021). Several reviews, while predating the most recent findings, are useful for understanding the key regions of the components (Marston and Zamora, 2020; Cheng and Regnier, 2016). The latter paper includes a useful diagram of the troponin components showing the position of many mutations along the primary structure.

### The CB Region of TnT is Localized Near Actin-Tropomyosin in the Inactive State

FRET measurements showed that in forming the inactive-Ca^2+^-free state from the inactive-Ca^2+^-bound state, CB residues 275 and 289 approach tropomyosin-190 (Johnson et al., 2019; Zhu et al., 2022). Residue 275 was closer to tropomyosin-190 than residue 289 of TnT.

These FRET results as well as the effects of truncating residues from the C-terminal region of TnT suggest that the C-terminal 14 amino acids of human cardiac TnT are important for positioning tropomyosin in the inactive state. The C-terminal region of TnT likely binds to actin or tropomyosin in order to hold tropomyosin in the inactive state (or bind to a position that blocks tropomyosin movement into the active states). Other data suggests that a site in the C-terminal region of TnT binds to tropomyosin near Cys 190 under conditions of very low free Ca^2+^ (Morris and Lehrer, 1984). That second tropomyosin binding site of TnT was reported to be between residues 197 and 239 of human cardiac TnT (Jin and Chong, 2010), or within the terminal 31 residues (Pearlstone and Smillie, 1981), or within the terminal 17 residues (Tanokura et al., 1983).

Further FRET studies showed that Ca^2+^ binding to TnC caused the CB region of TnT to move away from actin as well as from tropomyosin (Zhu et al., 2022). That is, there appears to be a coordinated movement of regions of TnT and TnI away from actin-tropomyosin upon Ca^2+^ binding. The extent of movement of the CB region away from actin-tropomyosin increased when Ala replaced the basic residues within the CB region of TnT. This further movement reflects the shift from about 30% occupancy of the active state to 70% occupancy under these conditions.

### The Location of the CB Region of TnT in the Active State

Ca^2+^ binding to the regulatory site(s) of TnC opens a hydrophobic pocket to which the switch region of TnI can bind (Herzberg and James, 1985) but it was unclear whether this was sufficient to release the inhibitory region of TnI from actin. There is an indication that the N-lobe of TnC is able to nudge the inhibitory region of TnI off of actin (Tobacman, 2021) allowing the switch region of TnI to bind to the hydrophobic pocket of TnC.

The CB region of TnT might also bind near the N-lobe of TnC in the active state (Johnston et al., 2019). However, FRET measurements show that the CB region of TnT remains far from TNC at both low and high free Ca^2+^ levels (Zhu et al., 2022). Because of the potential importance of the CB region in regulation, this discrepancy must be resolved.

### The CB Region of TnT is Critical for Normal Changes in TnI Interactions

Removal of the basic residues of the CB region of TnT caused the switch and inhibitory regions of TnI to move away from actin-tropomyosin in the same manner observed with Ca^2+^ binding to wild type regulated actin filaments. Formation of the inactive-Ca^2+^-bound state requires both the inhibitory region on TnI (Van Eyk and Hodges, 1988) and the long C-terminal region of TnI (residues 164–210), known as the mobile region (Ramos, 1999), (Wong et al., 2019). Now it appears that the CB region of TnT is also required to position tropomyosin into the inhibitory position. The ribbon diagram of the CB region of TnT in Figure 1 illustrates the possible change in position of the CB region of TnT in going from the relaxed to the active, high Ca^2+^-state.

The basic amino acid residues in the C-terminal region of TnI also appear to be critical for forming the inactive-Ca^2+^-free state. Charge replacements near the end of the IT helix (TnI-R145G and TnT-R278C) increased the resting force and increased the Ca^2+^ sensitivity of fibers (Brunet et al., 2014). Furthermore, these effects of the I and T mutations on the inactive state were additive.

We mentioned earlier that an inactive-Ca^2+^-bound state forms even in the virtual absence of Ca^2+^ when wild type TnT is replaced with Δ14 TnT or HAHA TnT. Although that seems to be a contradiction of terms that idea is clarified by FRET studies. Actin filaments containing HAHA TnT at low free Ca^2+^, would have the inhibitory region of TnI (light blue in Figure 1) and the CB region of TnT (blue and black dashed curve) positioned away from actin tropomyosin as they are for wild type filaments at saturating Ca^2+^. The switch region of TnI is also detached from actin but is not bound to the hydrophobic pocket of TnC as opening of the hydrophobic pocket of TnC is a rare event in the absence of bound Ca^2+^.

## Furure Prospects

Investigations of mutations of troponin have shown that having a population of regulated actin filaments with too little Ca^2+^ activation, too much Ca^2+^ activation or with too little difference between the inactive and active states (stabilization of the intermediate state) all result in cardiomyopathies. This observation complicates treatment of cardiomyopathies as it is critical to establish the natural balance. For example, the Ca^2+^-sensitizer Bepridil does increase activity at low free Ca^2+^ levels but it inhibits activity at high Ca^2+^ levels and seems to stabilize the inactive intermediate state of the actin filament (Varughese et al., 2011).

The many naturally occurring mutants of troponin as well as post-translationally modified forms of troponin provide opportunities to uncover additional details of actin-based regulation of contraction. The C-terminal region of TnT is an added region of TnT that limits Ca^2+^ activation providing additional activation possibilities upon binding of activating or force producing states of myosin. This region may be a future target for therapies. The function of the CB region of TnT is not entirely understood. Although this region is closer to actin-tropomyosin in the relaxed state than in the active state, we do not know if it is bound to either actin or tropomyosin. Even more uncertainty exists over the location of the CB region of TnT in the Ca^2+^ active state and in the fully active state.

The CB region of TnT reduces basal activity of regulated actin and limits Ca^2+^ activation in solution and decreases Ca^2+^ sensitivity in muscle fibers. Binding of force producing myosin crossbridges to actin restores full activation. This CB region is common in higher animal forms and its preservation suggests that this dual regulation by Ca^2+^ and binding of “activating” forms of myosin to actin is an advantage to the striated muscles of these organisms. It is unclear, at present, what that advantage is. A full understanding of this question requires knowledge of how all of the other modulators of contraction (phosphorylation, C protein etc.) operate together. Fortunately, troponin mutants may contribute to answering these and other questions.
